# Commentary “Recent advances in circadian rhythms in cardiovascular system”

**DOI:** 10.3389/fphar.2015.00132

**Published:** 2015-06-26

**Authors:** Stephane Fournier, Olivier Muller

**Affiliations:** Department of Cardiology, University of Lausanne Hospital CenterLausanne, Switzerland

**Keywords:** myocardial infarction, STEMI, circadian rhythm, circadian clocks, coronary disease

We read the recent publication by Chen and Yang with interest (Chen and Yang, 2015). In this review of excellent quality, the authors detail our current understanding of the links between circadian rhythms and cardiovascular system. Nevertheless, several important aspects of many recent key-findings have been overlooked in the Section “Circadian Rhythms and Myocardial Infarction.”

In fact, the authors focus on the well-documented higher rate of myocardial infarction occurring in the morning and on the circadian variation of cardiac functions related to the heart remodeling. From our point of view, they fail to discuss various recent important publications based on robust data supporting a link between circadian rhythms and myocardial infarction size and death from myocardial infarction.

Indeed, different clinical studies recently reported circadian variations of ischemic burden in patients with acute ST-elevation myocardial infarction (STEMI). In Reiter et al. (2012) and our team (Fournier et al., 2012) observed higher peak creatine kinase (CK) activity (as a proxy for myocardial infarction size) in patients with symptoms onset occurring between 00:00 and 05:59. Accordingly, these data indicate a shift between hour of maximal occurrence (in the second part of the morning) and time of maximal severity.

In 2013, differences in terms of vulnerability of the cardiomyocyte to ischemia were studied in 1021 patients undergoing elective percuaneous coronary intervention (PCI) between 2007 and 2011 (Fournier et al., 2014). Patients were divided into two groups according to the starting time of the PCI: the morning group (*n* = 651) between 07:00 and 11:59, and the afternoon group (*n* = 370) between 12:00 and 18:59. The rate of periprocedural myocardial infarction was statistically lower in the morning group compared to the afternoon group (20% vs. 30%, *p* < 0.001). This difference remained statistically significant after propensity score matching (21% vs. 29%, *p* = 0.03).

Finally, in 2014 and 2015, two important studies by Mahmoud et al. (2014) and by our team (Fournier et al., 2015) confirmed the initial results based on STEMI patients. From a multicenter registry of 6799 consecutive STEMI patients undergoing primary PCI between 2004 and 2010, Mahmoud et al. observed that infarct size exhibited circadian variation with largest infarct size in patients with symptom onset around 3 in the morning (estimated peak CK 1322 U/l; 95% confidence interval (CI): 1217–1436) and smallest infarct size around 11:00 in the morning (estimated peak CK 1071 U/l; 95% CI: 1001–1146; relative reduction 19%; *p* = 0.001). In our data based on 6223 STEMI patients admitted to 82 acute-care hospitals in Switzerland and treated with PCI within 6 h of symptom onset, only a 24-h harmonic was significantly associated with peak CK (*p* = 0.0001). The maximum average peak CK value (2315 U/L) was for patients with symptom onset at 23:00, whereas the minimum average (2017 U/L) was for onset at 11:00. In these studies, the relation between myocardial infarction size and hour at symptom onset was independent of ischemic time (time from symptoms onset to revascularization) or myocardial infarction management.

Furthermore, different data suggest that patients with symptom onset occurring by night have higher rate of mortality during their hospitalization or even at follow-up. In our first publication (Fournier et al., 2012) based on 353 consecutive patients with STEMI treated by PCI, 30-day mortality for STEMI patients with symptom onset occurring between 00:00 and 05:59 was significantly higher than in any other time group (*p* < 0.05). In our second study based on the 6223 patients, 23 patients (3.58%) died during their hospitalization and only the 24-h harmonic was significantly associated with in-hospital mortality. The risk of death from STEMI was highest for patients with symptom onset at 00:00 and lowest for those with onset at 12:00.

Thus, the evidence of higher vulnerability of the heart to ischemia when a myocardial infarction occurs by night is now robust and based on a large number of studies. These data confirm recent observations based on animal models where cardiomyocyte circadian clock affected cardiac responses to various stressors, including ischemia/reperfusion, by modulating multiple cardioprotective signaling pathways (Durgan and Young, 2010; Durgan et al., 2010). Indeed, in the study of Durgan et al. (2010), myocardial infarctions occurring during the sleep-to-wake transition time were 3.5 times larger than those occurring between the mid activity time but deletion of the clock gene abolished these differences, proving a central role of the circadian molecular machinery.

## Conflict of interest statement

The authors declare that the research was conducted in the absence of any commercial or financial relationships that could be construed as a potential conflict of interest.
